# Impaired myogenic response of the afferent arteriole contributes to the increased susceptibility to renal disease in Milan normotensive rats

**DOI:** 10.14814/phy2.13089

**Published:** 2017-02-13

**Authors:** Ying Ge, Fan Fan, Sean P. Didion, Richard J. Roman

**Affiliations:** ^1^ Department of Pharmacology and ToxicologyUniversity of Mississippi Medical CenterJacksonMississippi

**Keywords:** Chronic kidney disease, diabetic nephropathy, glomerulus, renal hemodynamics

## Abstract

Milan normotensive (MNS) rats are more susceptible to the development of renal disease than Milan hypertensive (MHS) rats, but the genes and pathways involved are unknown. This study compared the myogenic response of isolated perfused afferent arterioles (Af‐Art) and autoregulation of renal blood flow (RBF) and glomerular capillary pressure (Pgc) in 6–9‐week‐old MNS and MHS rats. The diameter of the Af‐Art of MHS rats decreased significantly from 14.3 ± 0.5 to 11.5 ± 0.6 *μ*m when perfusion pressure was elevated from 60 to 120 mmHg. In contrast, the diameter of Af‐Art of MNS rats did not decrease. RBF was well autoregulated in MHS rats, but it increased by 26% in MNS rats. Pgc rose by 11 mmHg when renal perfusion pressure (RPP) was increased from 100 to 140 mmHg in MNS but not in MHS rats. Protein excretion increased from 10 ± 1 to 245 ± 36 mg/day in MNS rats as they aged from 3 to 11 months but it did not increase in MHS rats. We also compared the development of proteinuria in MNS and MHS rats following the induction of diabetes with streptozotocin. Protein excretion rose from 16 ± 3 to 234 ± 43 mg/day in MNS rats, but it remained unaltered in MHS rats. These data indicate that the myogenic response of the Af‐art is impaired in MNS rats and increased transmission of pressure to the glomerulus may contribute to renal injury in MNS rats similar to what is seen in fawn‐hooded hypertensive and Dahl salt‐sensitive rats.

## Introduction

Hypertension and diabetes are the leading causes of end‐stage renal disease (ESRD), accounting for over 67% of the cases (Collins et al. 2009). The Medicare costs for the treatment of the 500,000 patients with ESRD exceed $62 billion/year (Collins et al. 2009). However, only about half the patients with hypertension or diabetes ever develop chronic kidney disease, and very little is known about the genes and pathways that determine susceptibility.

Bianchi et al. (1974b) first reported on the development of a new genetic rat model of hypertension termed the Milan hypertensive strain (MHS) along with a normotensive control strain (MNS). They were created by selective inbreeding of Wistar rats with the highest and lowest systolic pressures for many generations. MHS start to develop hypertension between 3 and 6 weeks of age, and this is associated with greater sodium retention than that seen in age‐matched MNS rats (Bianchi et al. 1975). Subsequent renal transplantation studies indicated that the kidney plays an important role in the development of hypertension in this strain (Bianchi et al. 1974a).

Baer and Bianchi reported that glomerular filtration rate (GFR) and single nephron GFR are lower in 3–5‐week‐old MHS than in MNS rats prior to the development of hypertension (Baer et al. 1978). On the other hand, others reported that GFR is higher in 3–5‐week‐old MHS than MNS rats and tubuloglomerular feedback (TGF) responsiveness is nearly absent at this time (Boberg and Persson 1986). Tubuloglomerular feedback responsiveness is enhanced during the development of hypertension in 5–7–week‐old MHS rats, and GFR is reduced following volume expansion due to a failure to reset TGF (Boberg and Persson 1986). These authors suggested that these alterations in renal hemodynamics may promote sodium retention and trigger the development of hypertension (Boberg and Persson 1986). GFR is not significantly different in adult MNS and MHS after the development of hypertension (Baer et al. 1978). However, glomerular capillary pressure (Pgc) is elevated by 5–7 mmHg, so overall glomerular filtration area or the ultrafiltration coefficient must be reduced in MHS rats (Baer et al. 1978). Despite the increase in Pgc, the kidney of hypertensive MHS is largely protected from the development of proteinuria and glomerulosclerosis, perhaps due to an elevated renal vascular resistance (Brandis et al. 1986; Pugliese et al. 2007a), enhanced tubuloglomerular feedback responsiveness (Boberg and Persson 1986), and/or vascular hypertrophy of the preglomerular arteries (Brandis et al. 1986). In contrast, MNS rats develop progressive proteinuria, glomerulosclerosis, and renal interstitial fibrosis as they age in the absence of systemic hypertension or diabetes (Brandis et al. 1986; Floege et al. 1997; Kriz et al. 1998; Menini et al. 2004). MNS rats also are more susceptible to the development of proteinuria and glomerular disease following the induction of diabetes with streptozotocin (STZ) (Pugliese et al. 2005, 2007). In contrast, STZ‐treated MHS rats like Sprague–Dawley and SHR rats are resistant to the development of proteinuria (Kaizu et al. 1995; Carlstrom et al. 2015). The mechanisms underlying the susceptibility of MNS rats, and the resistance of the MHS strain to the development of renal disease remain poorly understood.

Our laboratory has demonstrated that the myogenic response of afferent arteriole (Af‐Art) and autoregulation of renal blood flow (RBF) are impaired in fawn‐hooded hypertensive (FHH) (Williams et al. 2011; Burke et al. 2013) and Dahl salt‐sensitive (S) rats (Williams et al. 2008; Ge et al. 2014) that develop proteinuria and focal glomerulosclerosis as they age and following the development of hypertension. Dahl S rats are also far more susceptible to the development of diabetic nephropathy than Sprague–Dawley or other strains of rats (Korner et al. 1997; Slaughter et al. 2013).

We have also reported that the impaired myogenic response in FHH rats is associated with an increase in the large conductance potassium channel activity in renal vascular smooth muscle cells (Burke et al. 2013). Transfer of a region of chromosome 1 containing 15 genes including the gamma‐adducin or adducin3 (Add3) gene from BN rats rescued the myogenic response of the Af‐Art, autoregulation of RBF and attenuated the development of renal injury (Burke et al. 2013) in an FHH.1^BN^ congenic strain. We also identified a sequence variant in the coding region of the Add3 gene in FHH rats relative to other strains (Burke et al. 2013). In our preliminary work, we recognized that this same variant was reported in MNS versus MHS rats, although it was originally assumed that MHS rats carried the mutant allele (Bianchi et al. 2005). Since Add3 is a candidate gene for the development of renal disease in FHH rats, and MNS rats share the same variant in Add3 as FHH rats, this study tested whether an impairment in the myogenic response of the Af‐Art may be associated with the susceptibility of MNS to develop proteinuria and renal injury as they age and following induction of diabetes with STZ.

## Methods

### General

Experiments were performed on 53 male MHS and 57 MNS rats obtained from breeding colonies maintained at the University of Mississippi Medical Center. The breeders were a gift from Dr. Allen Cowley at the Medical College of Wisconsin who derived his colony from MNS and MHS rats provided by Dr. Giuseppe Bianchi from the University of Milan. All protocols were approved by the Institutional Animal Care and Use Committee, and are consistent with the NIH Guide for the Care and Use of Laboratory Animals.

### Comparison of the myogenic response of the Af‐Art of MNS and MHS rats

These experiments were performed on 6–9‐week‐old MHS and MNS rats. Briefly, the kidneys were removed and sliced along the corticomedullary axis. A single superficial Af‐Art with an attached glomeruli was microdissected and transferred to a temperature‐controlled chamber mounted on an inverted microscope. The Af‐Art was cannulated with concentric glass pipettes and incubated at 37°C in Minimum Essential Media (MEM, Sigma, St. Louis, MO) as we have previously described (Burke et al. 2013; Ge et al. 2014). Perfusion pressure was maintained at 60 mmHg. After a 30‐min equilibration period, the inner diameter of the Af‐Art was recorded. Then, the pressure was increased to 120 mmHg, and the diameter of the vessel was redetermined.

### Comparison of autoregulation of renal blood flow and glomerular capillary pressure in MNS and MHS rats

These experiments were performed on 9–12‐week‐old MNS and MHS rats. The rats were anesthetized with ketamine (30 mg/kg, i.m.) and thiobutabarbitol (50 mg/kg i.p.). The femoral artery and vein were cannulated for the measurement of mean arterial pressure (MAP) and the rats received an *i.v*. infusion of 2% BSA in a 0.9% NaCl solution at a rate of 100 *μ*L/min. Ties were placed around the mesenteric and celiac arteries and an adjustable clamp positioned around the aorta to allow for control of renal perfusion pressure (RPP). An ultrasound flow probe (Transonic System, Ithaca, NY) was placed on the left renal artery to measure RBF. After surgery and a 30‐min equilibration period, MAP was increased by tying the celiac and mesenteric arteries. Then, RPP was adjusted back to 100 mmHg using a clamp on the aorta above the renal arteries. After a 15‐min equilibration period, the RBF response to a step increase in RPP from 100 and 140 mmHg was recorded.

### Measurement of glomerular capillary pressure

Experiments were performed to access autoregulation of Pgc by measuring stop flow pressures in the proximal tubule of MNS and MHS rats as we previously described (Williams et al. 2011; Ge et al. 2014). The rats were prepared as described above with ties on the mesenteric and celiac artery and a clamp on the aorta to control RPP. The pressure was adjusted to 100 mmHg and stop flow pressure was measured in proximal tubules using a servo‐null pressure device (model 900, World Precision Instruments, Brandon, FL) after blocking tubular flow by microinjecting a high vacuum grease (Apiezon grease Type T, Apiezon products M&I materials LTD, Manchester, UK). Then, RPP was increased to 140 mmHg, and Pgc was recorded from the same tubules sampled during the control period.

### Effect of aging on protein excretion in MNS and MHS rats

These experiments were performed on different groups of 3‐, 5‐, 7‐, 9‐, and 11‐month‐old MNS and MHS rats (six rats per strain at each time point). The rats were housed in a metabolic cage overnight to measure protein excretion. Samples of blood were collected from the 3‐ and 11‐month‐old animals to measure plasma creatinine concentration. The kidneys of the 11‐month animals were collected and fixed in a 10% buffered formalin solution. Paraffin sections were prepared and stained with Masson's trichrome to determine renal cortical and medullary fibrosis (percentage blue staining). The degree of glomerulosclerosis was measured on 30 glomeruli per section as previously described (Williams et al. 2008, 2011; Burke et al. 2013).

### Comparison of the development of proteinuria in STZ‐treated MNS and MHS rats

These experiments were performed on six MHS and nine MNS rats that were 8–weeks‐old. The catheter of a telemetry unit (TA11PA‐C40; Data Sciences International, St. Paul, MN) was implanted in the femoral artery, and the tip advanced to the junction with the aorta for measurement of MAP. The unit was then implanted in a pocket created under the skin of the abdomen. After a 1 week recovery period, baseline MAP was recorded for 3 h a day between 9 and 12 are on three consecutive days. Diabetes was induced by a single injection of STZ (50 mg/Kg, i.p.), and a long‐acting insulin implant (2 U/day, Linshin Canada, Ontario, Canada) was implanted subcutaneously to maintain blood glucose levels between 400 and 500 mg/dL. MAP was measured at 3 week intervals throughout the experiment. At each point, urine was collected overnight for measuring protein excretion and blood was collected from the tail vein for measuring blood glucose and HbA1C levels.

### Measurement of RBF and GFR

After completion of the chronic study, the rats were anesthetized with ketamine (30 mg/Kg) and thiobutabaritol (50 mg/Kg). Catheters were implanted in the femoral artery for the measurement of MAP and the femoral vein for an intravenous infusion of a 2% BSA solution containing FITC‐labeled inulin (2 mg/mL; Sigma) in a 0.9% NaCl solution at a rate of 6 mL/h. An ultrasonic blood flow probe was placed around the left renal artery. After a 30‐min equilibration period, urine and plasma samples were collected during two 30‐min collection periods for measurement of urine flow and electrolyte excretion, and GFR, MAP, and RBF were recorded. At the end of the experiment, a blood sample was collected to measure plasma electrolyte and creatinine concentrations. Then, the kidneys were flushed with saline and collected for histology to determine the degree of glomerular injury and renal fibrosis as described above.

### Statistical analysis

Mean values ± SEM are presented. The significance of differences in control and experimental values within the same animal was determined by a paired *t*‐test. The significance of differences in corresponding values between groups was determined by an unpaired *t*‐test or an ANOVA followed by Holm–Sidak test. A value of *P* < 0.05 was considered to be significant.

## Results

### Comparison of the myogenic response of the Af‐Art of MNS and MHS rats

**Figure 1:**
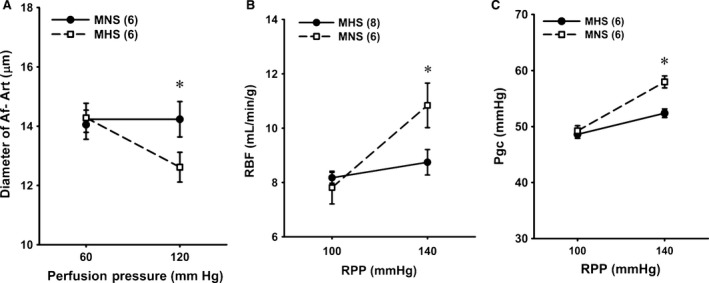
Comparison of the myogenic response of afferent arterioles. (A) and autoregulation of renal blood flow (RBF) (B) and glomerular capillary pressure (Pgc) (C) in MNS and MHS rats. Numbers in parentheses indicate the number of vessels, tubules or rats studied. *Indicates a significant difference from the corresponding value within a strain. MHS, Milan hypertensive; MNS, Milan normotensive; RBF, renal blood flow.

A comparison of the myogenic response in the Af‐Art of MNS and MHS rats is presented in Figure 1A. The diameter of the Af‐Art was decreased from 14.3 ± 0.5 to 12.5 ± 0.6 *μ*m in response to an elevation of perfusion pressure from 60 to 120 mmHg in MHS rats, but it was not significantly altered in MNS rats.

### Comparison of autoregulation of RBF and Pgc in MNS and MHS rats

Autoregulation of RBF was intact in the MHS rats, and RBF was not significantly altered when RPP was increased from 100 to 140 mmHg (Fig. 1B). In contrast, RBF increased from 7.8 to 10.8 mL/min/g in MNS rats when the pressure increased over this range (Fig. 1B), and the autoregulatory index averaged 0.9. Pgc rose by 9 mmHg in MNS rats when RPP was increased from 100 to 140 mmHg. It was well autoregulated and did not increase significantly in MHS rats (Fig. 1C).

### Time course of changes in protein excretion and renal injury in MNS and MHS rats

**Figure 2:**
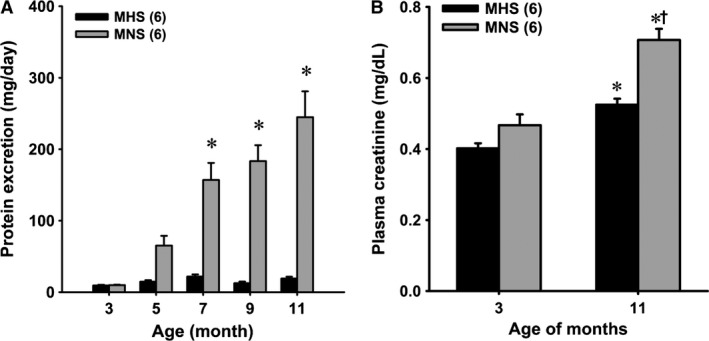
Panel A – Comparison of the time course of the development of proteinuria in MNS and MHS rats. Panel B – Comparison of plasma creatinine concentrations in 3‐ and 11‐month‐old MNS and MHS rats. Numbers in parentheses indicate the number of rats studied at each time point. *Indicates a significant difference from the corresponding value within a strain. ^†^Indicates a significant difference from the corresponding value in MHS rats. MHS, Milan hypertensive; MNS, Milan normotensive.

Protein excretion was not significantly different in 3‐ and 5‐ month‐old MNS and MHS rats (Fig. 2A). Protein excretion increased from 10 ± 1 to 245 ± 36 mg/day in MNS rats as they aged from 3 to 11 months, but it did not increase significantly in MHS rats. Plasma creatinine concentration was similar in 3‐month‐old MNS and MHS rats. Plasma creatinine concentration rose in both MNS and MHS rats as they aged, however, the increase was greater in MNS than in MHS rats. At 11 months of age, plasma creatinine concentration was 40% higher in MNS than in MHS rats (Fig. 2B).

**Figure 3:**
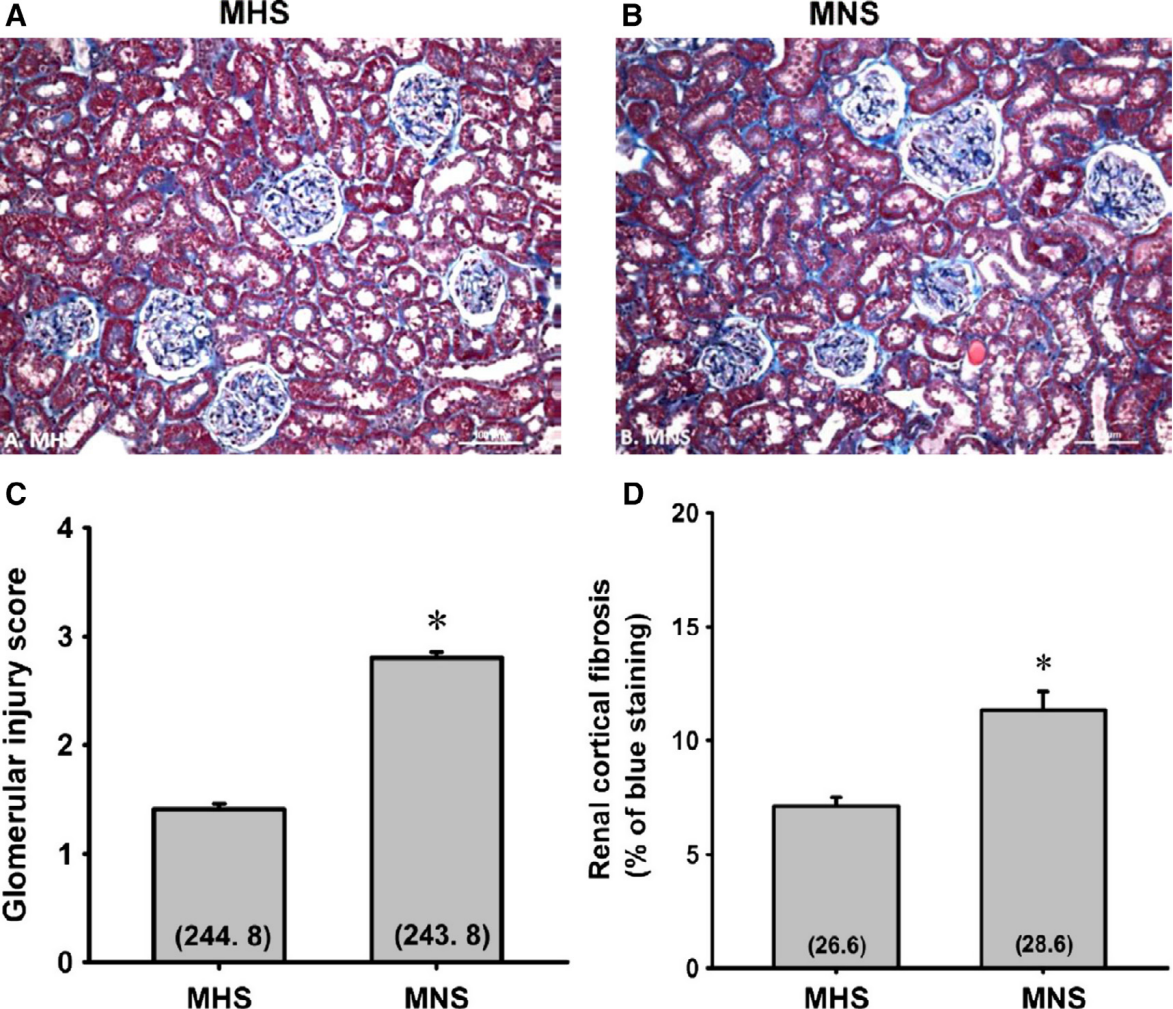
Comparison of the renal histopathology (A and B), the degree of glomerulosclerosis (C), and renal cortical fibrosis (D) in the kidneys of 11‐month‐old MNS and MHS rats. Numbers in parentheses indicate the number of glomeruli or cortical areas scored per number of rats studied. *Indicates a significance difference from the corresponding value in MHS rats. MHS, Milan hypertensive; MNS, Milan normotensive.

A comparison of the degree of glomerulosclerosis and renal cortical fibrosis in 11‐month‐old MNS and MHS rats are presented in Figure 3. The kidneys of the 11‐month‐old MNS rats exhibited moderate focal glomerulosclerosis characterized by hypertrophy of 20–30% of the glomeruli, thickening of glomerular basement membrane, expansion of the mesangial matrix, narrowing of glomerular capillaries and increased glomerular and renal interstitial fibrosis (Fig. 3A and B). The glomerular injury and cortical fibrosis scores were significantly greater in MNS than age‐matched MHS rats (Fig. 3C and D).

**Figure 4:**
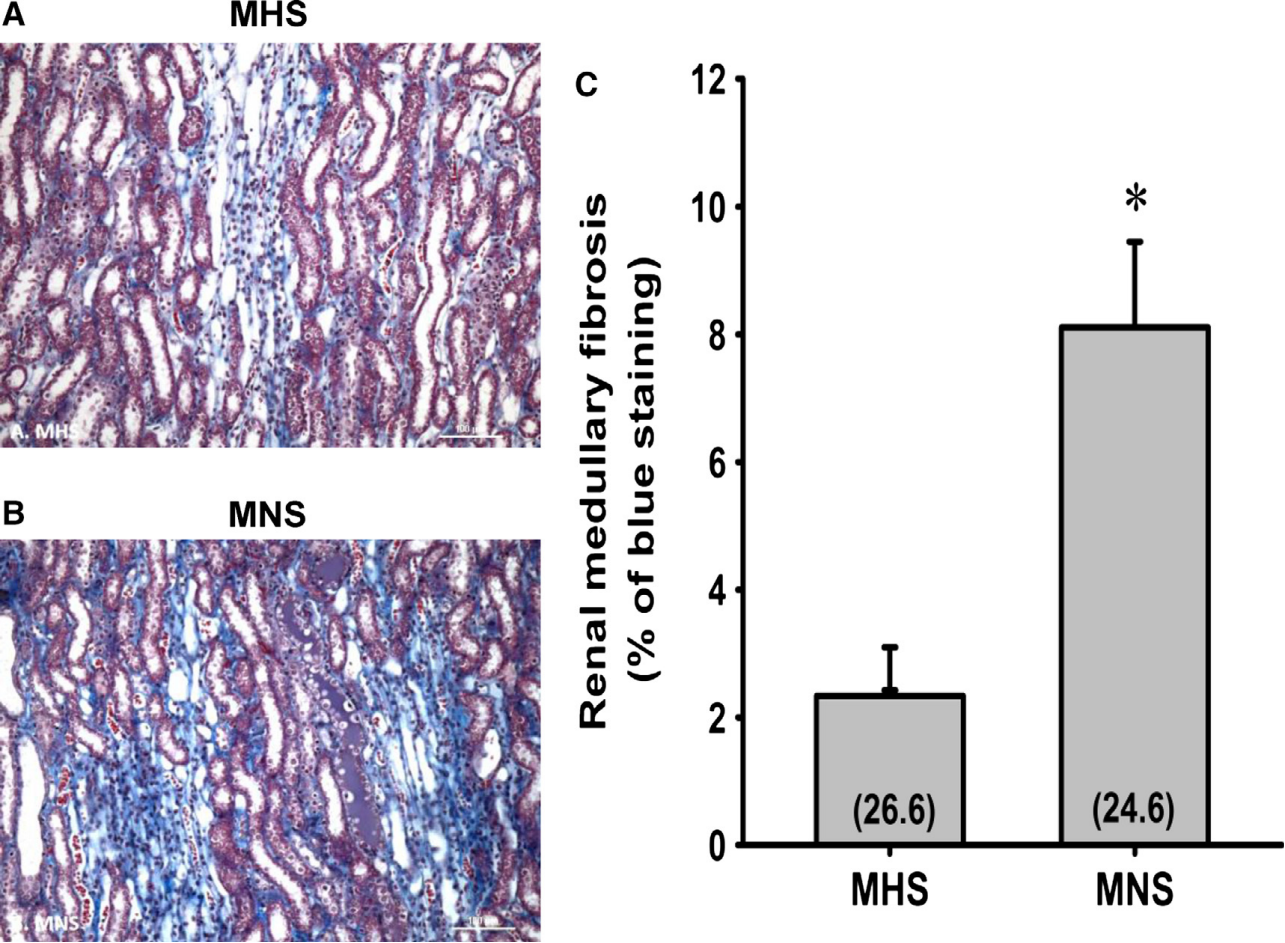
Comparison of the renal histopathology (A and B), the degree of renal outer medullary fibrosis (C) in 11‐month‐old MNS and MHS rats. Numbers in parentheses indicate the number of areas/rats studied per group. *Indicates a significance difference from the corresponding value in MHS rats. MHS, Milan hypertensive; MNS, Milan normotensive.

The degree of renal fibrosis in the outer medulla of 21‐week‐old MHS and MNS rats are presented in Figure 4. MNS rats exhibited a greater fibrosis (Fig. 4B) (blue staining) especially around the vasa recta vascular bundles than that seen in MHS rats (Fig. 4A). The percentage of medullary area stained blue with Mason's trichrome was fourfold greater in MNS than in MNS rats (Fig. 4C). MNS rats exhibited more tubular necrosis and tubular casts (purple staining) than in aged‐match MHS rats (Fig. 4A).

### Comparison of the development of proteinuria and renal injury in MNS and MHS rats after the induction of diabetes with STZ

**Figure 5:**
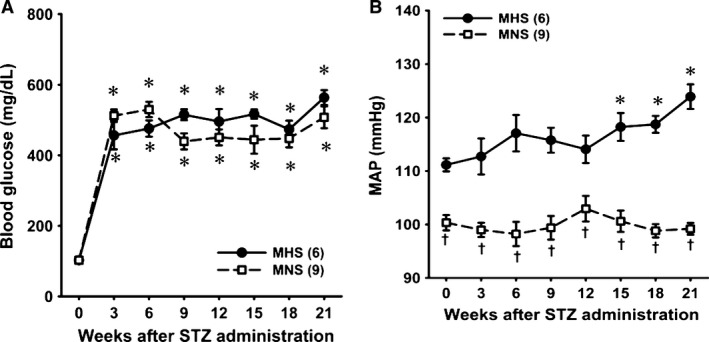
Time course of changes in blood glucose levels (A) and mean arterial pressure (MAP) (B) after induction of diabetes with streptozotocin in MNS and MHS rats. MAP was recorded for 3 h a day between 9 and 12 am. *Indicates a significant difference from the corresponding value within a strain. ^†^Indicates a significant difference from the corresponding value in MHS rats. MHS, Milan hypertensive; MNS, Milan normotensive.

Baseline blood glucose levels were similar in MNS and MHS rats before injection of STZ (Fig. 5A). Blood glucose levels increased to a similar extent in STZ‐treated MNS and MHS rats. Control MAP was 11 mmHg higher in 9‐week‐old MHS than MNS rats (Fig. 5B). MAP increased by another 12 mmHg over the course of the study in MHS rats but remained unaltered in MNS rats.

**Figure 6:**
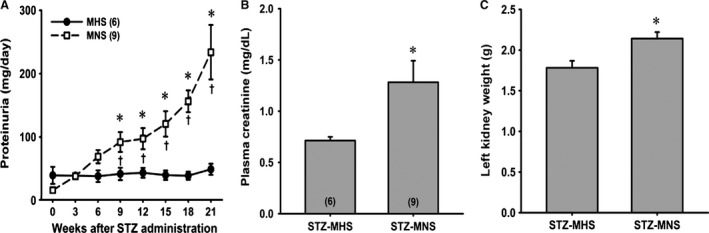
Time course of the development of proteinuria in MNS and MHS rats following the induction of diabetes with streptozotocin (Panel A). Comparison of plasma creatinine concentration (B) and left kidney weight (C) 21 weeks following the induction of diabetes in MNS and MHS rats. Numbers in parentheses indicate the number of rats studied at each time point. *Indicates a significant difference from the corresponding value within a strain. ^†^Indicates a significant difference from the corresponding value in MHS rats. MHS, Milan hypertensive; MNS, Milan normotensive.

Proteinuria was not significantly different in 9‐week‐old MNS and MHS rats (Fig. 6A). Proteinuria increased significantly 9 weeks after injection of STZ in MNS rats, but it was not significantly altered in MHS rats. It continued to increase progressively over the course of the experiment. Plasma creatinine concentration was significantly higher at the end of the study in STZ‐MNS rats than in age‐matched STZ‐MHS rats (Fig. 6B). Kidney weight, an index of renal hypertrophy, was also significantly greater in MNS than MHS rats (Fig. 6C).

**Figure 7:**
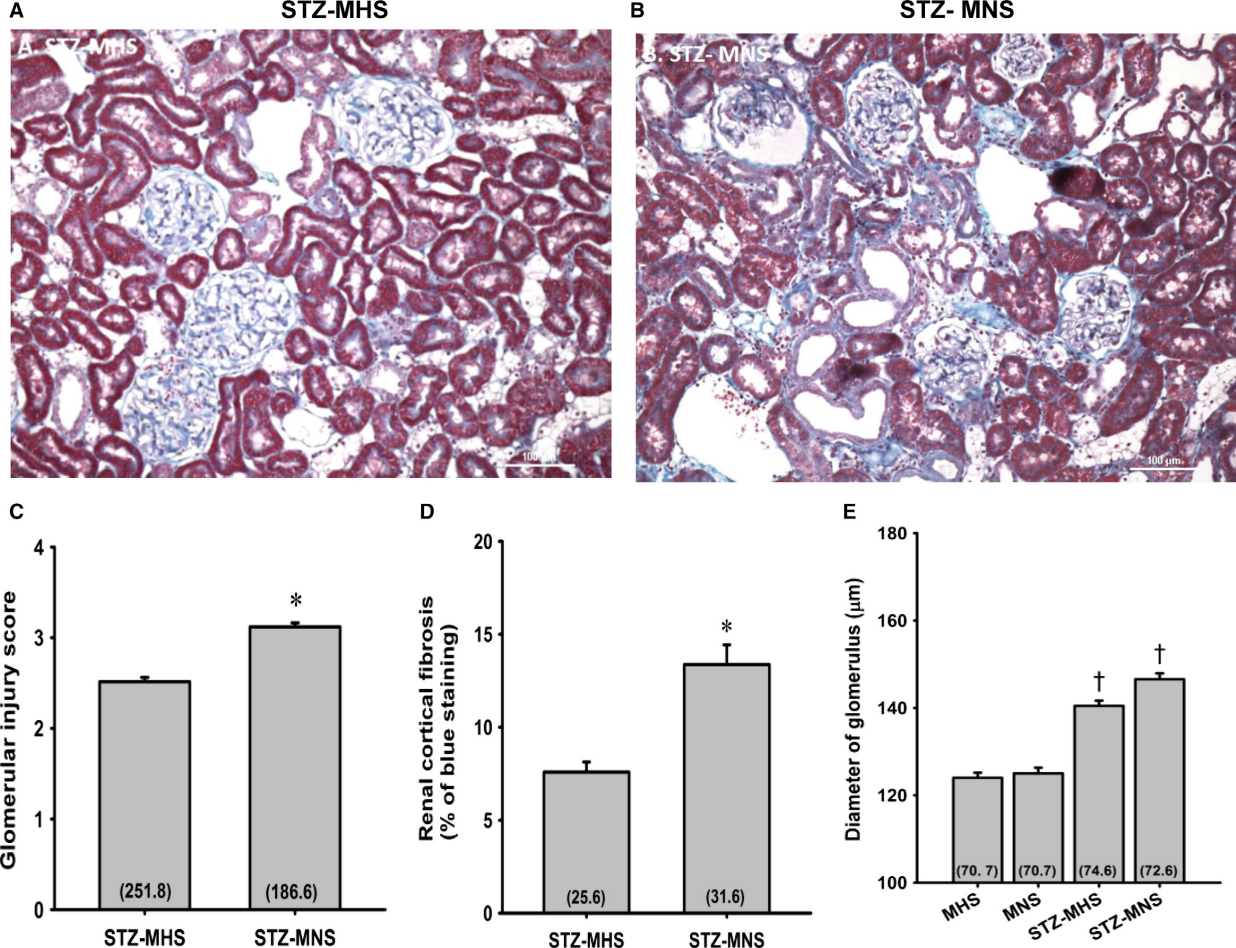
Comparison of the renal histopathology (A and B), the degree of glomerulosclerosis (C), and renal cortical fibrosis (D) 21 weeks following the induction of diabetes with streptozotocin (STZ) in MNS and MHS rats. Comparison of the diameters of glomerular in MNS, MHS, STZ‐MHS, and STZ‐MNS rats (E). Numbers in parentheses indicate the number of glomeruli or cortical areas scored per number of rats studied. *Indicates a significance difference from the corresponding value in MHS rats. ^†^Indicates a significant difference from values measured in nondiabetic age‐matched control rats. MHS, Milan hypertensive; MNS, Milan normotensive; STZ, streptozotocin.

**Figure 8:**
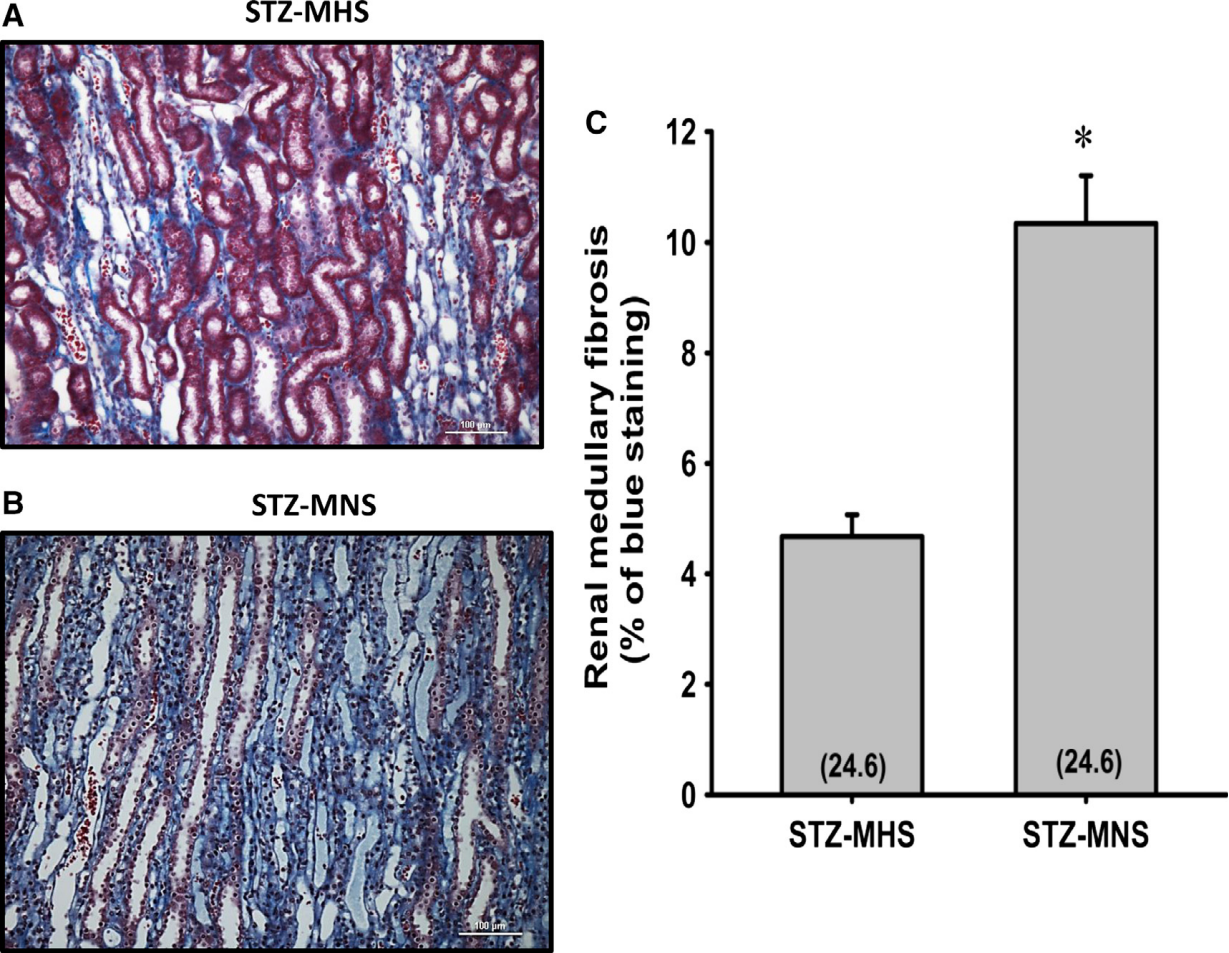
Comparison of the renal outer medullary histopathology (A and B), and the degree of renal outer medullary fibrosis (C) 21 weeks following the induction of diabetes with streptozotocin (STZ) in MNS and MHS rats. Numbers in parentheses indicate the number of areas/rats studied per group. *Indicates a significance difference from the corresponding value in MHS rats. MHS, Milan hypertensive; MNS, Milan normotensive; STZ, streptozotocin.

The degree of renal hypertrophy and injury in STZ‐MNS and STZ‐MHS rats is presented in Figure 7. The expansion of the glomerular mesangial matrix, glomerular and renal interstitial fibrosis and glomerular injury scores were significantly higher in MNS than MHS rats (Fig. 7A, B, C, and D). Similarly, the degree of fibrosis and tubular necrosis in the outer medulla was greater in MNS than MHS rats 21 weeks after induction of diabetes (Fig. 8A, B, and C). Glomerular diameter increased significantly in diabetic MNS and MHS rats treated with STZ (Fig. 7E).

A summary of the renal clearance results obtained from MNS and MHS rats, 21 weeks after the induction of diabetes, is presented in Table 1. Body weights were similar in the diabetic MNS and MHS rats. Urine flow, potassium excretion, RBF, and GFR were not significantly different in diabetic MNS an MHS rats. Sodium excretion was significantly increased in STZ‐treated MNS than MHS rats.

**Table 1:** Summary of the renal clearance results obtained from MNS and MHS rats 21 weeks after the induction of diabetes

	STZ‐MHS (*n* = 6)	STZ‐MNS (*n* = 9)
MAP (mmHg)	127.2 ± 3.5	111.4 ± 4.5^a^
RBF (mL/min)	10.7 ± 1.4	12.9 ± 0.8
GFR (*μ*L/min)	2.0 ± 0.3	2.6 ± 0.5
Urine flow rate (*μ*L/min)	35.3 ± 4.5	39.5 ± 7.8
Sodium excretion (nEq/min)	564.4 ± 70.5	1434.2 ± 314.9^a^
Potassium excretion (nEq/min)	2977.6 ± 368.5	2447.2 ± 471.3

a Indicates a significance difference from the corresponding value in MHS rats. GFR, glomerular filtration rate; MAP, mean arterial pressure; MHS, Milan hypertensive; MNS, Milan normotensive; RBF, renal blood flow.

## Discussion

Previous studies have indicated that MNS rats develop rather severe glomerulosclerosis and proteinuria in the absence of hypertension and diabetes by 9–12 months of age, whereas MHS are largely resistant (Bianchi et al. 1974b; Brandis et al. 1986; Pugliese et al. 2005). However, the genes and mechanisms involved remain to be determined. In this study, we investigated whether the myogenic response of isolated perfused Af‐Art and autoregulation of RBF is impaired in MNS rats similar to what has been reported in FHH (Simons et al. 1993; Williams et al. 2011; Burke et al. 2013), Brown Norway (BN) (Wang et al. 2000) and Dahl S rats (Takenaka et al. 1992; Karlsen et al. 1997; Williams et al. 2008) that are susceptible to renal disease.

The present results are similar to those reported previously (Brandis et al. 1986; Floege et al. 1997; Kriz et al. 1998). The MNS rats developed significant proteinuria by about 5 months of age and exhibited severe proteinuria, focal glomerulosclerosis, and renal interstitial fibrosis at 11 months of age. The age‐matched MHS rats did not exhibit any increase in proteinuria and exhibited much less glomerular injury and renal interstitial fibrosis. Plasma creatinine concentration was also significantly higher in 11‐month‐old MNS than MHS rats. We also found that MNS rats developed proteinuria, renal and glomerular hypertrophy, focal glomerulosclerosis and renal interstitial fibrosis following the induction of diabetes with STZ. In contrast, the MHS rats were resistant to the development of diabetic‐induced proteinuria and renal injury. The time course of the development of proteinuria and renal injury in our diabetic MNS rats is very similar to that reported by others (Pugliese et al. 2005, 2007). They concluded that MHS rats might be resistant to the development of diabetic nephropathy due to vascular hypertrophy that prevents transmission of pressure to the glomerulus (Pugliese et al. 2005, 2007). However, renal hemodynamics was not accessed in the previous studies, and there was no time control data. In this study, we found that the time course of the development of proteinuria following the induction of diabetes in MNS rats closely paralleled the age‐related changes in proteinuria seen in this strain (Fig. 6A vs. 2A). The degree of proteinuria and renal injury 21 weeks after the induction of diabetes in the 30‐week‐old MNS rats were not significantly different from that seen in the 7–9‐month‐old (24–36‐week‐old) MNS rats in the aging study. The only difference was that glomerular size was about 30% greater in the diabetic MNS rats than their age‐matched controls. Moreover, we found that the glomeruli were hypertrophied to the same extent in diabetic MHS rats that did not develop proteinuria. Thus, we cannot draw any definite conclusions as to whether the proteinuria seen in STZ‐treated MNS rats is due to diabetic nephropathy or simply a consequence of the typical increase in proteinuria observed in this strain with aging.

Previous studies have concluded that the hypertrophy of the renal vasculature (Brandis et al. 1986; Menini et al. 2004) and elevated tubuloglomerular feedback responsiveness (Boberg and Persson 1986) that prevents transmission of pressure to the glomerulus may provide protection from hypertension‐induced renal injury in MHS rats similar to what is seen in Spontaneous Hypertensive rats (Loutzenhiser et al. 2006; Burke et al. 2014; Carlstrom et al. 2015). However, in this study, the inner diameter of the Af‐art was similar in 6–9‐week‐old MHS and MNS rats (Fig. 1A), and there were no obvious vascular hypertrophy or changes in wall thickness in MHS rats. MAP was 12 mmHg higher in 9‐weeks‐old MHS than MNS rats (Fig. 5B), but the MHS rats were not very hypertensive at this age as MAP only averaged 112 mmHg. Thus, the magnitude and duration of hypertension at this early age was probably not sufficient to produce vascular hypertrophy or remodeling. There has been little consideration that the increased susceptibility of the MNS strain to renal injury might be related to changes in renal hemodynamics that increase transmission of pressure to the glomerulus similar to what has been reported in Dahl S (Takenaka et al. 1992; Karlsen et al. 1997; Williams et al. 2008; Ge et al. 2014), BN (Wang et al. 2000) and FHH rats (Simons et al. 1993, 1994; Van Dokkum et al. 1999; Williams et al. 2011; Burke et al. 2013) as well as diabetic rats (Hostetter et al. 1982), deoxycorticosterone acetate‐salt (Hill and Heptinstall 1968; Moore et al. 1979; Deng and Schiffrin 1992) and reduced renal mass hypertensive rats (Bidani et al. 1987).

The results of this study indicate that the myogenic response is impaired in the Af‐Art of MNS rats, while it is intact in MHS rats. Since the rapid autoregulation of RBF and Pgc to elevations in arterial pressure is mainly mediated via the myogenic response, we examined whether the changes in vascular reactivity in MNS rats also affected autoregulation of RBF and Pgc in response to a step increase in RPP. We found that autoregulation of RBF was impaired in MNS rats, and it rose by 39% in response to a 40% increase in RPP from 100 to 140 mmHg. More importantly, the rise in systemic pressure was transmitted to the glomerulus, and Pgc increased by 20% when RPP was increased by 40% in MNS rats. In contrast, autoregulation was intact in MHS, and RBF and Pgc were not significantly altered in response to the same stimulus. These results provide the first direct evidence that the myogenic response and autoregulation of RBF are impaired in MNS rats. The impaired response allows greater transmission of pressure to the glomerular capillaries in response to elevations in arterial pressure. Increased transmission of pressure increases pulsatile pressure and the distention of the glomerular capillaries that stimulates mesangial cells and podocytes to proliferate and increase matrix formation and fibrosis (Kriz et al. 1998; Loutzenhiser et al. 2006; Burke et al. 2014; Carlstrom et al. 2015). Pulsatile pressure also raises intracellular Ca^2+^ in podocytes that is thought to contribute to foot process effacement and loss of podocytes. In this regard, (Pugliese et al. 2007) has found evidence of glomerular barrier dysfunction in aged and diabetic MNS rats, but not in glomerulosclerosis‐resistant MHS rats. They suggested that the alterations in renal hemodynamics play an important role in the development of glomerulosclerosis and renal fibrosis in MNS rats, but this hypothesis was not directly studied.

Mutations in ADD1, which alter actin polymerization, disrupt the cytoskeleton and enhance Na^+^/K^+^‐ATPase activity and sodium transport in the kidney have been linked to the development of hypertension in man and MHS rats (Bianchi et al. 1994, 2005; Tripodi et al. 1996, 2004; Cusi et al. 1997). Variants in the Add3 genes have also been identified, but they are not associated with hypertension in MHS rats or man (Zagato et al. 2000; Cwynar et al. 2005). Adducin is a cytoskeletal protein consisting of *α*‐subunit (Add1) and *β*‐ (Add2) or *γ*‐ (Add3) subunits that regulate in actin–spectrin interactions. (Matsuoka et al. 2000) Add1 forms heterodimers with Add3 in most tissues including the cerebral and renal vasculature. Adducin plays an important role in the organization of the cytoskeleton, signal transduction, membrane trafficking, cell‐to‐cell contact formation, and cell migration (Matsuoka et al. 2000). Potentially, variants in either Add1 or Add3 could alter the cytoskeleton, trafficking of ion channels, and vascular reactivity. However, the roles of Add1 or Add3 in altering the myogenic response and vascular function, especially at the level of the middle cerebral artery (MCA) or Af‐Art, have not been directly studied. The results of this study indicate that the myogenic response of the Af‐Art and autoregulation of renal blood flow is impaired in MNS rats. Increased transmission of pressure to the glomerular capillaries may contribute to the development of proteinuria and glomerulosclerosis in MNS rats similar to what is seen in FHH and Dahl S rats in which autoregulation of RBF is also impaired (Takenaka et al. 1992; van Dokkum et al. 1999; van Rodijnen et al. 2002; Loutzenhiser et al. 2006; Burke et al. 2014; Carlstrom et al. 2015). Since MNS and FHH rats share a sequence variant in Add3, which is a positional candidate gene for the alterations in renal hemodynamics and proteinuria and renal disease in FHH rats (Mattson et al. 2007; Williams et al. 2011; Burke et al. 2013), it is tempting to speculate that it may play a similar role in MNS rats. However, further work is needed to test this hypothesis as it remains to be determined whether the shared K572Q mutation in the coding region of the Add3 gene in MNS and FHH rats relative to other strains (MHS, SHR, Dahl S rats) alters the function of this protein, its interactions with Add1, actin–spectrin interactions, and vascular tone.

In summary, the results of this study indicate that the myogenic response of Af‐Art and autoregulation of RBF is impaired in MNS rats. The resulting increase in the transmission of pressure to the glomerulus may contribute to the development of proteinuria and renal disease in this strain similar to what is seen in FHH and Dahl S rats in which autoregulation of RBF is also impaired.

## Conflict of Interest

None declared.
